# Supplementary material for: MERFISH+, a large-scale, multi-omics spatial technology resolves the molecular holograms of the 3D human developing heart
Source: bioRxiv. 2025 Nov 4:2025.11.02.686137. Preprint. [Version 1] doi: 10.1101/2025.11.02.686137 (PMC12637541; doi:10.1101/2025.11.02.686137)
Supplement: Supplement 2 [file NIHPP2025.11.02.686137v1-supplement-2.pdf]

bioRxiv preprint doi: https://doi.org/10.1101/2025.11.02.686137; this version posted November 4, 2025. The copyright holder for this preprint (which was not certified by peer review) is the author/funder, who has granted bioRxiv a license to display the preprint in perpetuity. It is made available under aCC-BY 4.0 International license.

Supplemental Figures

bioRxiv preprint doi: https://doi.org/10.1101/2025.11.02.686137; this version posted November 4, 2025. The copyright holder for this preprint (which was not certified by peer review) is the author/funder, who has granted bioRxiv a license to display the preprint in perpetuity. It is made available under aCC-BY 4.0 International license.

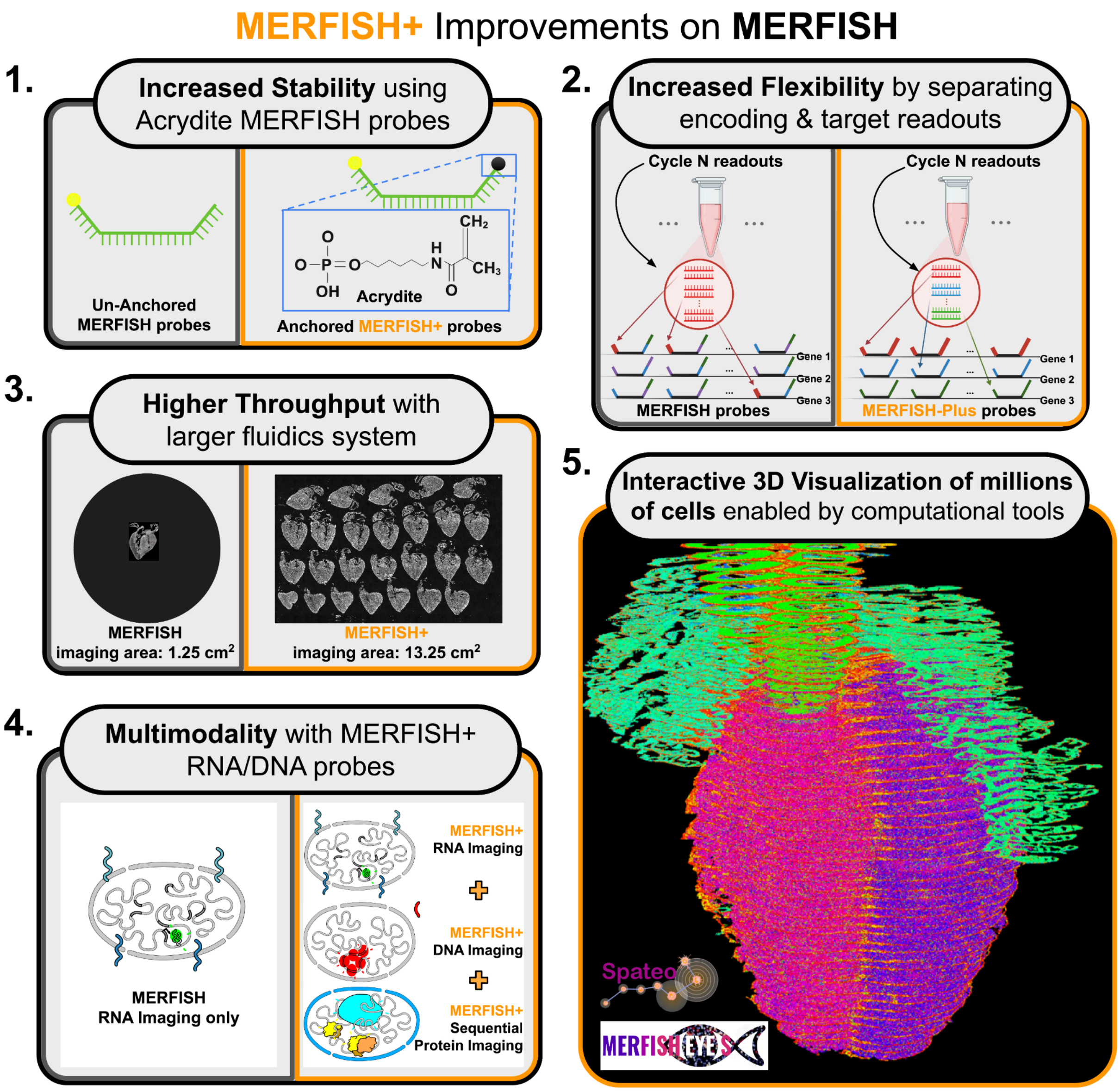

Figure S1. Schematic of the new MERFISH+ capabilities. 1. MERFISH+ probes are synthesized with a 5' acrydite modification to allow for their robust integration into protective hydrogels. This enhances the stability of experiments. 2. New barcoding strategy of MERFISH+ probes allows for a flexible scaling up of the number of genes profiled. 3. Higher throughput microscopy and microfluidics instrumentation allows for 10X higher imaging area with over 1.5 million cells profiled per experiment. 3. MERFISH+ probes facilitate multimodal and multiplexed imaging of RNA, DNA and histone marks while preserving the integrity of the signal for an extended time. 5. The higher throughput instrumentation, coupled with molecular alignment tools (Spateo-VI) allows for 3D reconstruction of entire human organs.

bioRxiv preprint doi: https://doi.org/10.1101/2025.11.02.686137; this version posted November 4, 2025. The copyright holder for this preprint (which was not certified by peer review) is the author/funder, who has granted bioRxiv a license to display the preprint in perpetuity. It is made available under aCC-BY 4.0 International license.

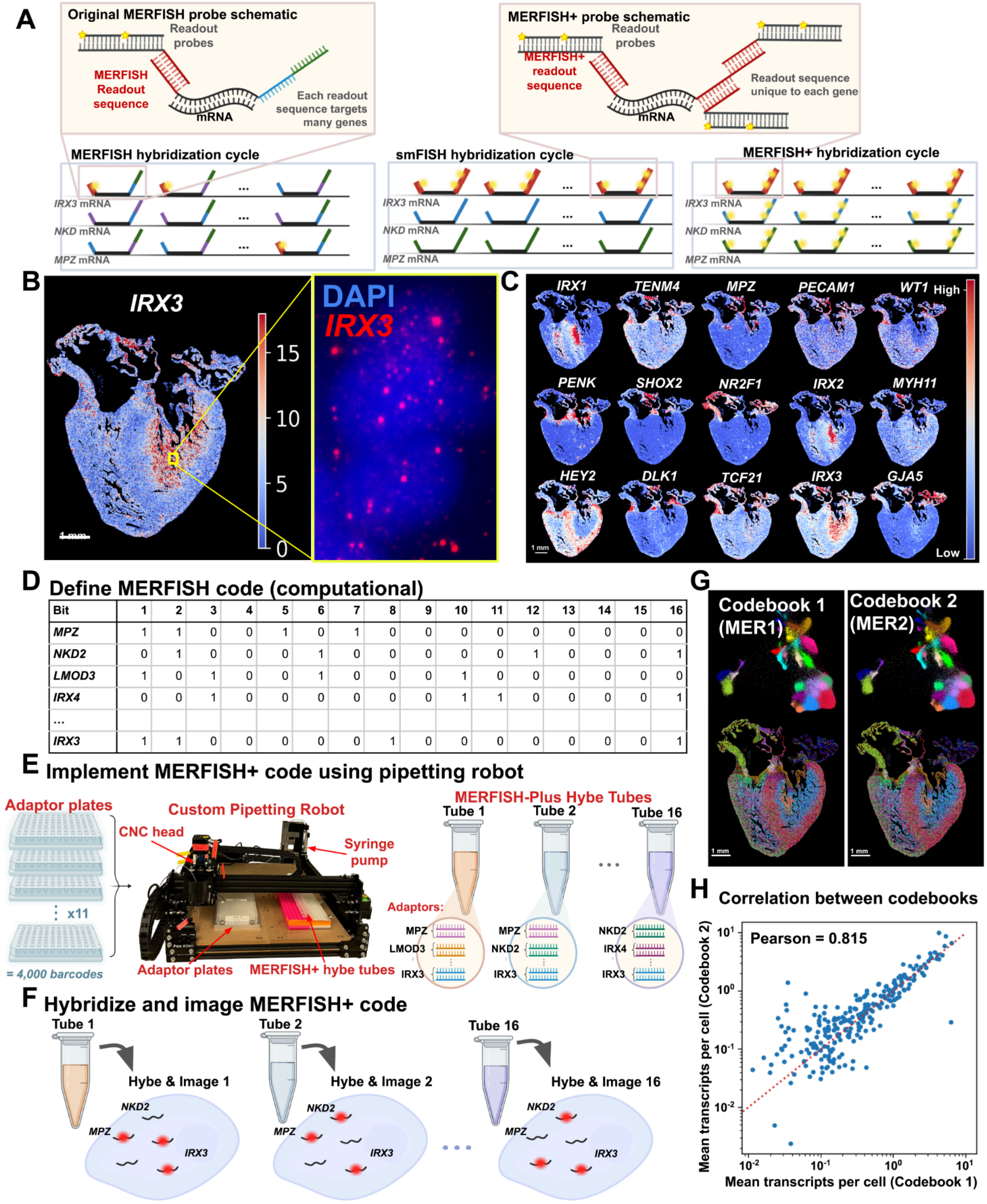

Figure S2. MERFISH+ probes allow for a flexible scaling up of the number of genes profiled. (A) The stability of the acrydite-conjugated probes, combined with stringent washing conditions, allow for a redesign of MERFISH probes (termed

bioRxiv preprint doi: https://doi.org/10.1101/2025.11.02.686137; this version posted November 4, 2025. The copyright holder for this preprint (which was not certified by peer review) is the author/funder, who has granted bioRxiv a license to display the preprint in perpetuity. It is made available under aCC-BY 4.0 International license.

MERFISH+ probes) in which the readout strategy of the original MERFISH design was changed for increased flexibility. The original MERFISH probe design (**A, top-left**) has built-in readout sequences targeting many different genes simultaneously in each hybridization cycle (**A, bottom-left).** This design does not enable individual genes to be imaged separately and if any mistakes were introduced during the design (i.e. the accidental inclusion of a gene with high expression), the MERFISH probes need to be reordered and resynthesized. In contrast, the MERFISH+ probe design added built-in unique readout sequences for all probes targeting each gene (**A, top-right**). This allows MERFISH+ probes for any gene or subset of genes to be readout using either through serial single-molecule (sm)-FISH or using MERFISH with an adaptive combinatorial readout strategy (**A, bottom-middle and bottom-right**). **(B) (C)** Single-molecule FISH images of selected genes from a 1,835 MERFISH library hybridized to a 16-um human developing heart section. **(D) (E) (F)** Schematic demonstrating that MERFISH+ probes facilitate designing and imaging custom combinatorial readout strategies for faster quantification of the genes of interest in each cell. An example MERFISH codebook designed for a set of ~250 genes optimized for the human heart is shown in (D). A custom pipetting robot (E) is then programmed to mix combinations of readout probes for each cycle of hybridization. The pipetting program corresponds to a predefined MERFISH combinatorial codebook such that, for instance, a value of 1 in columns 1,2,8 and 16 for gene *IRX3* (D) means that the readout probes for *IRX3* were pipetted and mixed for hybridization cycles 1,2,8 and 16 (F). **(G)** UMAPs and spatial distributions of cell types derived from two MERFISH experiments performed on the same sample and targeting the same genes with two different combinatorial codebooks. **(H)** Correlation of mean transcripts per cell between the two MERFISH experiments in (G) (Pearson's correlation coefficient of 0.815).

bioRxiv preprint doi: https://doi.org/10.1101/2025.11.02.686137; this version posted November 4, 2025. The copyright holder for this preprint (which was not certified by peer review) is the author/funder, who has granted bioRxiv a license to display the preprint in perpetuity. It is made available under aCC-BY 4.0 International license.

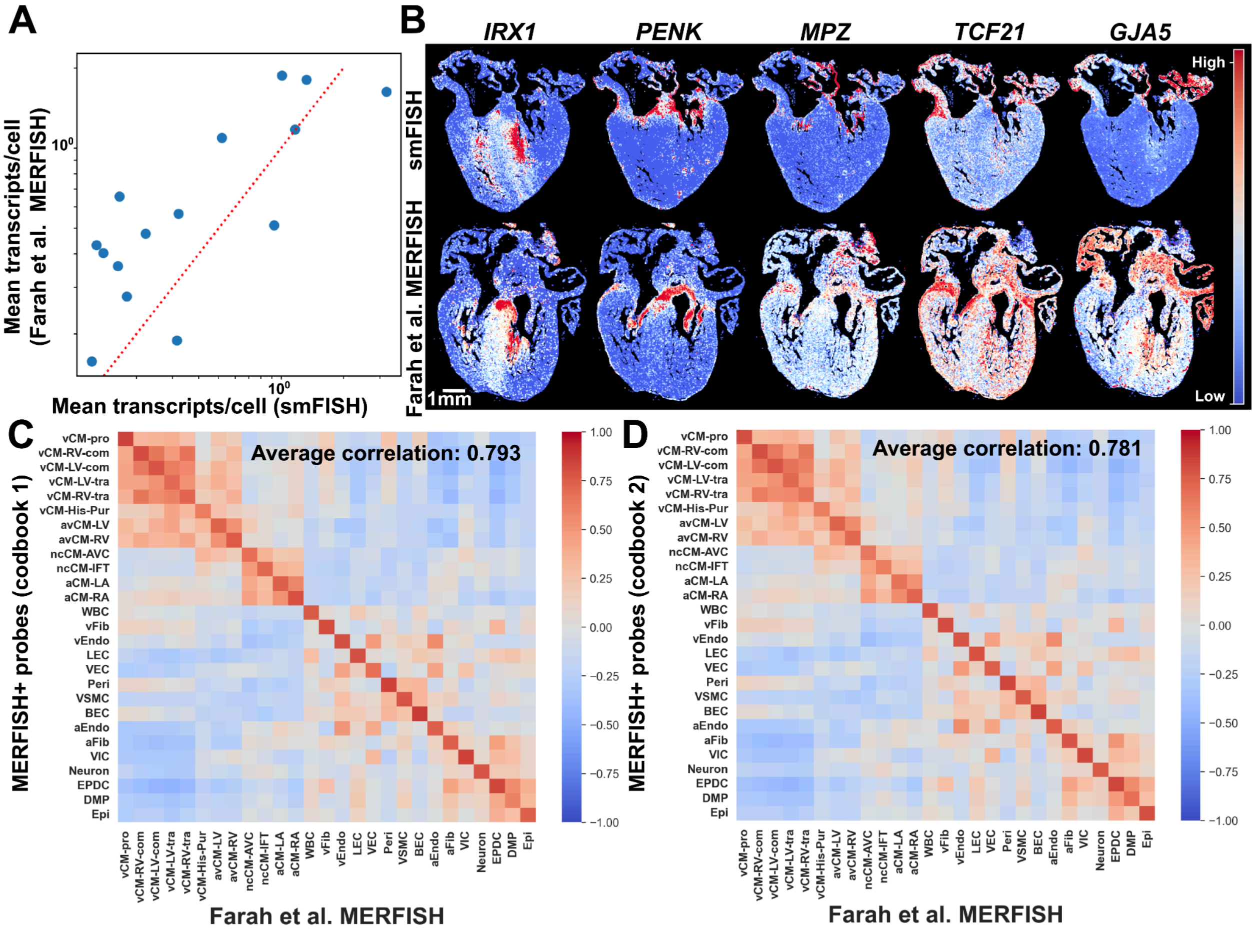

Figure S3. Assessing of MERFISH+ probe performance with prior MERFISH data. **(A)** Correlation of mean transcripts per cell between published MERFISH data[15] and single-molecule FISH data imaged with the new MERFISH+ probes (Pearson's correlation coefficient of 0.789). **(B)** Spatial distributions of gene expression per cell comparing published MERFISH data with single-molecule measurements of MERFISH+ probes. **(C, D)** Correlation matrices of average gene expression for 238 genes between MERFISH+ cell-type clusters and prior MERFISH clusters[15] across 2 codebooks in (C) and (D).

bioRxiv preprint doi: https://doi.org/10.1101/2025.11.02.686137; this version posted November 4, 2025. The copyright holder for this preprint (which was not certified by peer review) is the author/funder, who has granted bioRxiv a license to display the preprint in perpetuity. It is made available under aCC-BY 4.0 International license.

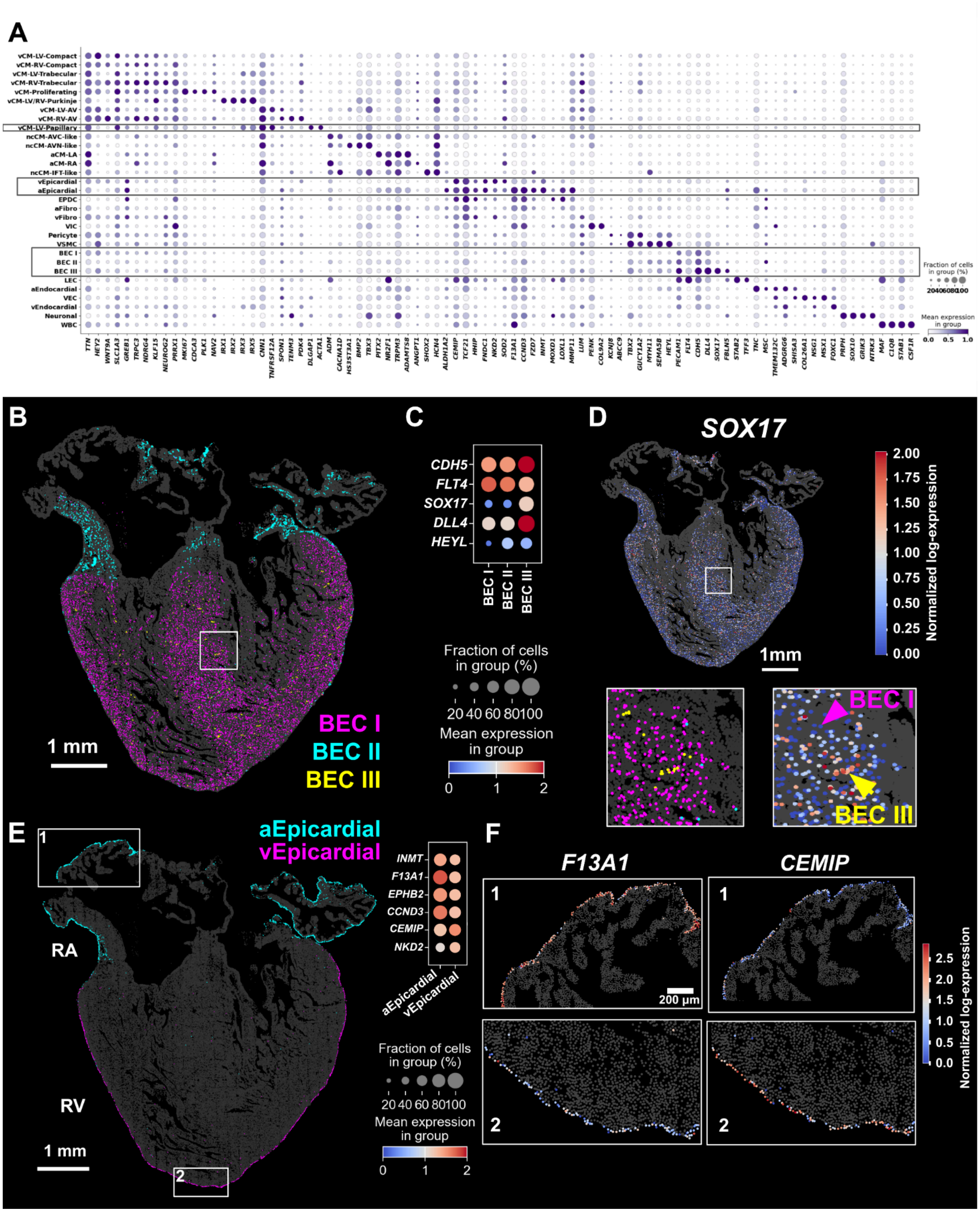

Figure S4. Spatial distribution and gene expression of new cell populations identified with 1,838 genes profiled. (A) Dot plot marking the differential gene expression across the cell types defined based on the 1,838 single-cell expression profiles. Newly identified cell populations are boxed. **(B)** Spatial distribution of three

bioRxiv preprint doi: https://doi.org/10.1101/2025.11.02.686137; this version posted November 4, 2025. The copyright holder for this preprint (which was not certified by peer review) is the author/funder, who has granted bioRxiv a license to display the preprint in perpetuity. It is made available under aCC-BY 4.0 International license.

BEC sub-populations (BEC I,II and III). **(C)** Gene expression of selected differentially expressed genes across BEC sub-populations. **(D)** Spatial gene expression of *SOX17*, enriched in BEC III cells. Insets show cell type definition of BECs and gene expression of *SOX17* for a zoomed in region of the heart marked by a white box. **(E)** Left: Spatial distribution of aEpicardial and vEpicardial cell populations. Right: Differential gene expression plot across aEpicardial and vEpicardial. **(F)** Spatial gene expression of *F13A1*, enriched in aEpicardial, and *CEMIP*, enriched in vEpicardial.

bioRxiv preprint doi: https://doi.org/10.1101/2025.11.02.686137; this version posted November 4, 2025. The copyright holder for this preprint (which was not certified by peer review) is the author/funder, who has granted bioRxiv a license to display the preprint in perpetuity. It is made available under aCC-BY 4.0 International license.

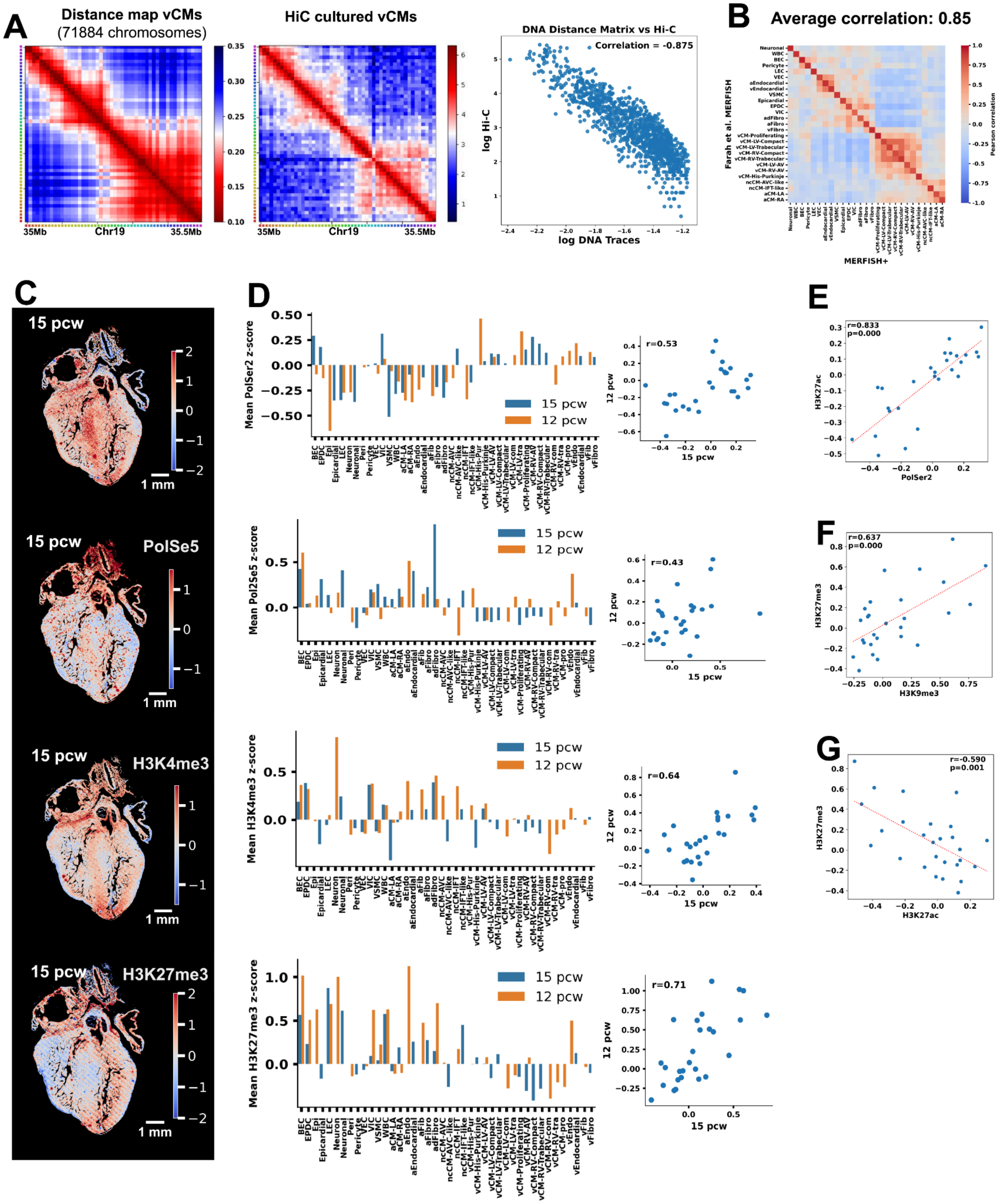

Figure S5. Validation of multimodal imaging results. (A) Left: Median distance map across vCMs of the 10kb segments comprising the FXYD locus (chr19:35Mb-35.5Mb) Middle: HiC data[41] for the FXYD locus in cultured differentiated cardiomyocytes. Right: Correlation of physical distance measured by chromatin tracing in vCMS and the contact probability measured by HiC (Pearson's correlation coefficient of 0.875). **(B)**

bioRxiv preprint doi: https://doi.org/10.1101/2025.11.02.686137; this version posted November 4, 2025. The copyright holder for this preprint (which was not certified by peer review) is the author/funder, who has granted bioRxiv a license to display the preprint in perpetuity. It is made available under aCC-BY 4.0 International license.

Correlation matrices of average gene expression for 238 genes in the multimodal MERFISH+ experiment cell-type clusters and prior MERFISH clusters[15] . **(C)** Spatial map of the z-scored antibody brightness for Pol2ser2, Pol2ser5, H3K4me3, H3K9me3 and H3K27me3 marks across cells in a 15 pcw human heart section. **(D)** Left: Bar plots of z-scored antibody brightnesses of the epigenetic marks in (C) across the cell types of two human heart sections (15 pcw - blue and 12 pcw -orange). Right: Correlation plots of the z-scored antibody brightnesses in (C) across the cell types of the two sections. **(E),(F),(G)** Correlation across cell types in a 15 pcw human heart section for z-scored brightnesses of H3K27ac vs Pol2ser2 (E), H3K27me3 vs H3K9me3 (F) and H3K27ac vs H3K27me3 (G). Pearson correlation coefficients are indicated.

bioRxiv preprint doi: https://doi.org/10.1101/2025.11.02.686137; this version posted November 4, 2025. The copyright holder for this preprint (which was not certified by peer review) is the author/funder, who has granted bioRxiv a license to display the preprint in perpetuity. It is made available under aCC-BY 4.0 International license.

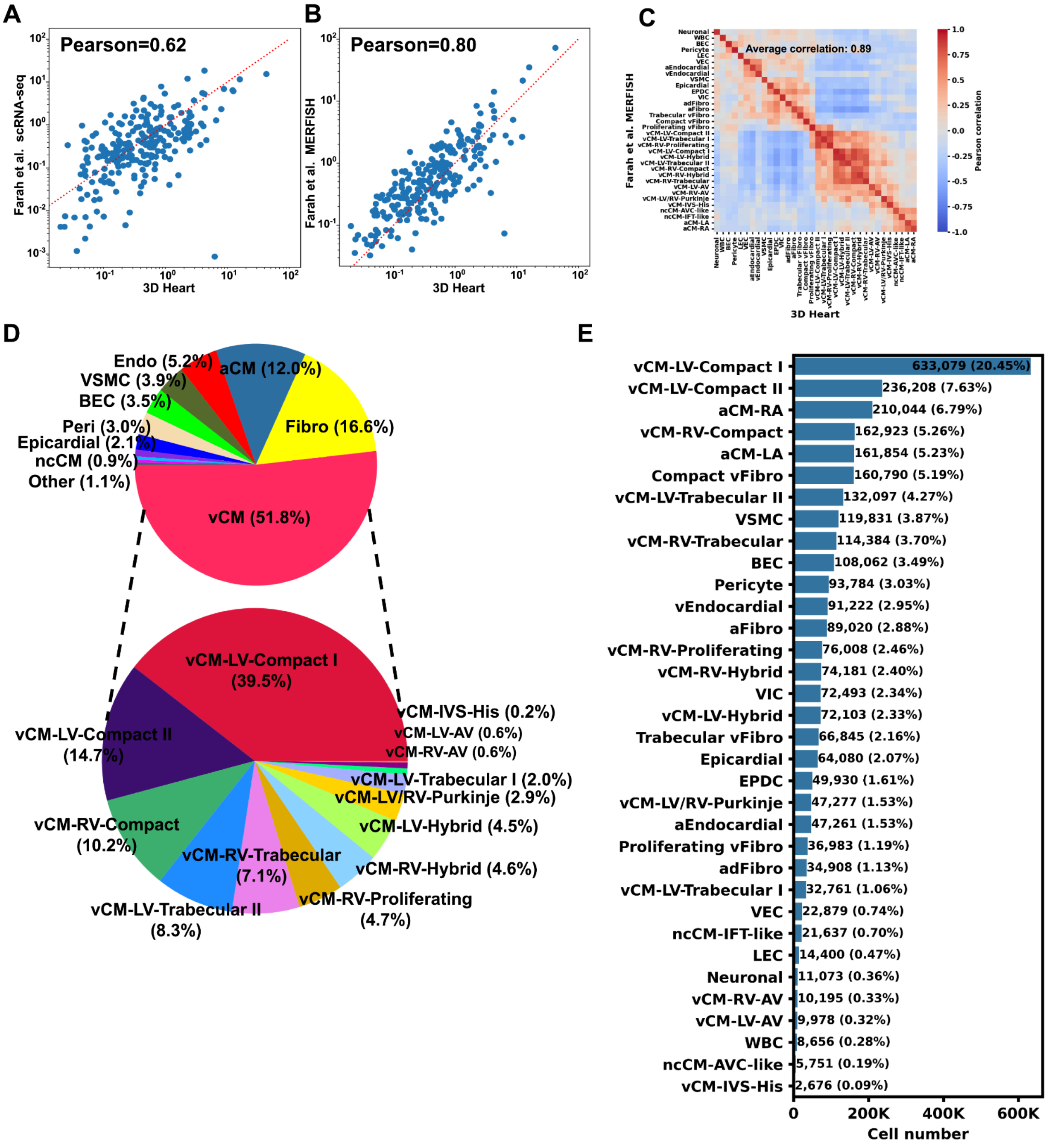

Figure S6. Cell type abundance across the 3D developing human heart (A),(B) Correlation of average transcripts per cell for each gene of the MERFISH+ 3D data with previously published snRNA-seq (A) and previously published MERFISH data (B). **(C)** Heatmap showing pairwise cell type to cell type correlation between this MERFISH data and the previously published MERFISH data. **(D)** Pie chart showing the proportions of major cell types in the heart (top) and the proportions of ventricular cardiomyocyte sub-types (bottom). **(E)** Bar plot with the percentage and number of cells of each cell type across the 3D heart.

bioRxiv preprint doi: https://doi.org/10.1101/2025.11.02.686137; this version posted November 4, 2025. The copyright holder for this preprint (which was not certified by peer review) is the author/funder, who has granted bioRxiv a license to display the preprint in perpetuity. It is made available under aCC-BY 4.0 International license.

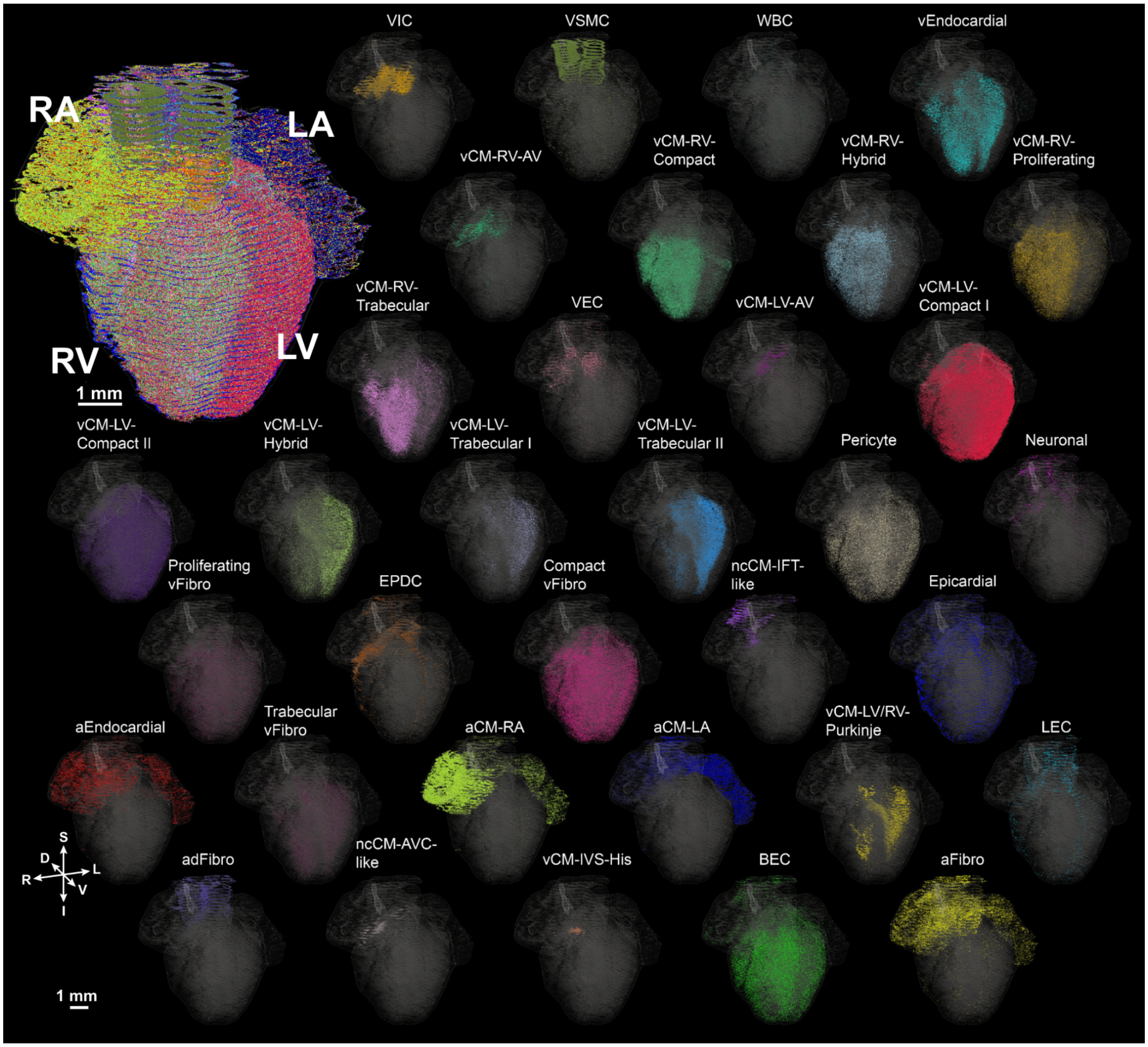

Figure S7. 3D spatial distribution of the cell types identified in the developing human heart. A composite image capturing all cell types is shown in the upper left corner. Each subpanel shows the 3D spatial distribution of one of the 34 cell populations identified.

bioRxiv preprint doi: https://doi.org/10.1101/2025.11.02.686137; this version posted November 4, 2025. The copyright holder for this preprint (which was not certified by peer review) is the author/funder, who has granted bioRxiv a license to display the preprint in perpetuity. It is made available under aCC-BY 4.0 International license.

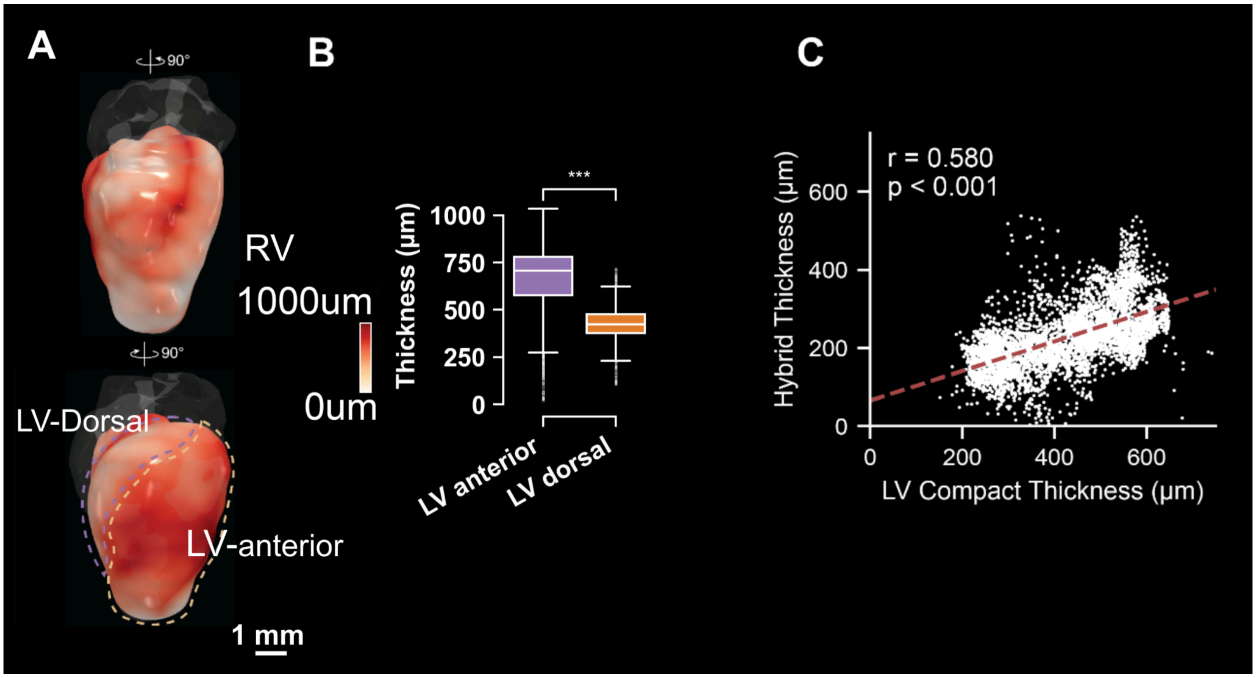

Figure S8. Quantification of the thickness of the ventricular free wall and the ventricular cardiomyocyte layers. (A) Thickness of the ventricular free wall measured from the reconstructed 3D heart, displayed across right and left views. The LV-anterior and dorsal, highlighted with dotted circles, where red surface indicates thicker areas and white indicates thinner areas. **(B)** Quantitative comparison of thickness in the LV-anterior and dorsal. *** indicates a p-value < 1e–3 using Student's t test. **(C)** Correlation of LV hybrid layer thickness and LV compact layer thickness. Pearson correlation coefficient (r) and p-value are indicated. Scale bars as indicated.

bioRxiv preprint doi: https://doi.org/10.1101/2025.11.02.686137; this version posted November 4, 2025. The copyright holder for this preprint (which was not certified by peer review) is the author/funder, who has granted bioRxiv a license to display the preprint in perpetuity. It is made available under aCC-BY 4.0 International license.

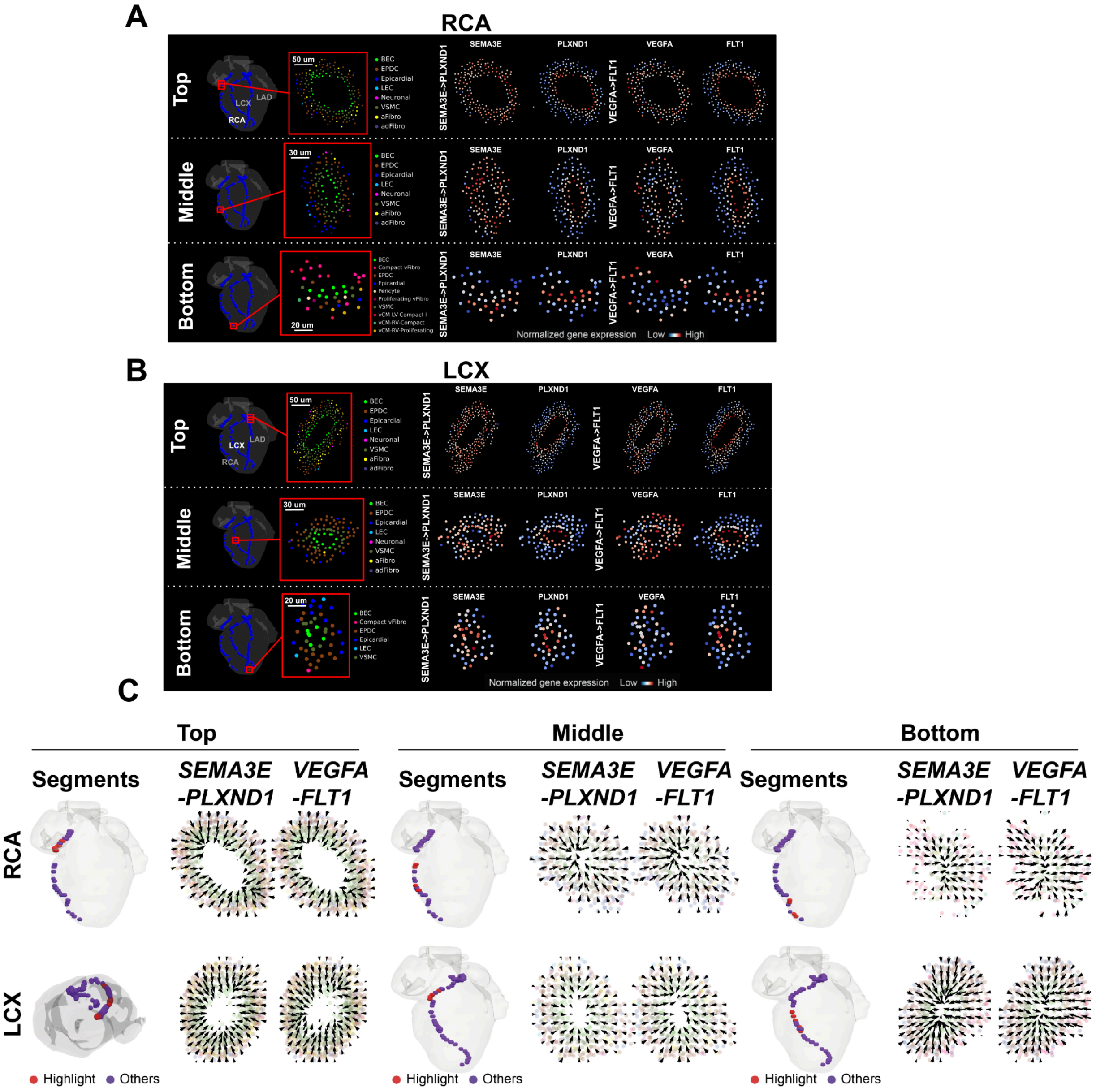

Figure S9. Semaphorin and VEGF signaling in the descending arteries. (**A**),**(B)** Cross-sections of the right coronary artery (RCA) (A) and the left circumflex artery (LCX) (B) showing cell types and imputed gene expression of two known ligand-receptor pairs *SEMA3E-PLXND1* and *VEGFA-FLT1*. (**C**) Spatial gradient of ligand-receptor interaction in cross-sections of the RCA (top) and LCX (bottom). Three segments, marked in red, along the RCA and LAD from the root to the distal tip are projected to create an aggregate representation of the cross-section. Vector fields (represented by quivers) show the direction and magnitude of *SEMA3E-PLXND1* and *VEGFA–FLT1* signaling flow in the LCX and RCA.
